# Efficacy and safety of low concentration hydrogen peroxide as nasopharyngeal lavage fluid in the treatment for nasopharyngeal carcinoma radiotherapy: a pilot cohort study

**DOI:** 10.7150/jca.83189

**Published:** 2023-04-02

**Authors:** Juan Xiong, Rong Liu, Yuying Zhang, Wenting Song

**Affiliations:** 1 Department of Radiation Oncology, Jiangxi Cancer Hospital, Nanchang City 330029, Jiangxi, China.; 2 Department of Medical Oncology, Nanchang People's Hospital, The Third Hospital of Nanchang City, Nanchang City 330002, Jiangxi, China.

**Keywords:** Nasopharyngeal Carcinoma, Nasopharyngeal Lavage, Hydrogen Peroxide, Nitrofurazone, Radiotherapy, Adverse Events

## Abstract

**Purpose:** This study aimed to evaluate and analyze the efficacy and safety of low concentration (0.15%) hydrogen peroxide as nasopharyngeal lavage fluid in the treatment for nasopharyngeal carcinoma radiotherapy.

**Methods:** Patients with nasopharyngeal carcinoma from Jiangxi cancer hospital were randomly divided into two cohorts. The training cohort (*n*= 50) received low concentration (0.15%) hydrogen peroxide as nasopharyngeal lavage fluid in the treatment for nasopharyngeal carcinoma radiotherapy, and the control cohort (*n*= 50) received 0.02% nitrofurazone lavage fluid. The primary endpoint of the study was result of short-term efficacy. Second endpoints were assessment of the linear visual analogue scale score and the incidences rate of nasopharyngeal radiation related toxicity.

**Results:** All patients had completed the scheduled nasopharyngeal radiotherapy except two patients in control cohort. The complete response, partial response, stable disease and disease objective response of nasopharyngeal primary tumor observed in the training cohort included 18 cases, 23 cases, 9 cases and 41 cases respectively, while in the control cohort 20 cases, 25 cases, 5 cases and 45 cases were recorded, respectively. The study showed a significant discrepancy in the incidence rate of radiation-related mucosa damage between the two. Specifically, Grade 1 and 2 included 37 cases (74.0%) in the training cohort, while in the control cohort the cases were 20 (40.0%). Grade 3 and 4 damage however reported an incidence of 26.0% and 60%, respectively, which clearly constitutes a significant statistical difference (P = 0.002). The assessment of linear visual analogue scale showed that the patients self-conscious comfortable feeling in the training cohort were significantly higher than in the control cohort (P = 0.003).

**Conclusions:** low concentration (0.15%) hydrogen peroxide as nasopharyngeal lavage fluid in the treatment for nasopharyngeal carcinoma patients is effective and safe, and can reduce nasopharyngeal local mucosa radiation related toxicity after radiotherapy.

## Introduction

Nasopharyngeal carcinoma (NPC) is a malignant tumor occurring in the nasopharynx. It is most common in Southeast Asian countries and Southern China. Radiotherapy plays a pivotal role in the treatment of NPC patients, as it significantly improves local control ratio and prolongs overall survival time 1-4. However, during radiotherapy, the nasopharynx and surrounding tissues are usually accompanied by hyperemia and edema of the membrane, necrosis and exfoliation of epithelial cells. Due to the resulting local bacterial multiplication, the target area can become inflamed. Failure to discharge the necrotic tissue of nasopharynx in a timely manner will affect the radiation dose distribution and the sensitivity of the cancer tissue to radiation, thus affecting the overall therapeutic efficacy 1, 5, 6. Consequentially, nasopharyngeal lavage is one of the most important auxiliary treatments for NPC radiotherapy 7. At the radiotherapy department of Jiangxi Cancer Hospital 0.02% nitrofurazone lavage fluid was once used to purge the nasopharynx. However, upon thorough examination, it was found that the nasopharynx still presented residual necrotic tissue post-purging. A local inflammation of the nasopharynx sometimes occurred with more serious its condition. The application of hydrogen peroxide (H_2_O_2_) was shown to have the effect of scavenging the necrotic tissue and anti-anaerobic bacteria 8, 9. However, the efficacy of H_2_O_2_ as nasopharyngeal lavage fluid for the auxiliary treatment with NPC patients has yet not been reported in the literature, and the correlation of H_2_O_2_ as nasopharyngeal lavage fluid for improving NPC radiotherapy efficacy and alleviating adverse events (AEs) of radiotherapy is still unclear. In this study, the principle of prospective, single-center, open-label, cohort study was adopted. The aim was to investigate the efficacy and safety of low concentration (0.15%) H_2_O_2_ as nasopharyngeal lavage fluid in the auxiliary treatment of NPC patients undergoing radiotherapy.

## Materials and methods

### Research design

This study was a randomized, controlled, prospective, and open-label cohort study (Trial Registration ID: 20185366). The patients were randomly divided into a training cohort and a control cohort. This study adopts random number table of simple randomization method. Participants in the first cohort would receive a low concentration (0.15%) H_2_O_2_ along with their radiation therapy, while participants belonging to the control cohort would instead undergo nitrofurazone lavage as an auxiliary treatment for their radiation therapy. The primary endpoints of the study were the disease objective response rate (ORR), complete response (CR), partial response (PR), progressive disease (PD) and stable disease (SD). The second endpoints were the assessment of the linear visual analogue scale (VAS) score and the incidences rate of nasopharyngeal radiation-related toxicity. This study was approved by Jiangxi cancer hospital research ethics committee.

### Patients eligibility and exclusion criteria

The inclusion eligibility criteria were as follows, (i) age > 18 and ≤ 70; (ii) eastern Cooperative Oncology Group (ECOG) ≤ 2; (iii) definitive histopathological diagnosis; (iv) the study time period was from the beginning of radiotherapy to three months after radiotherapy; (v) nasopharyngeal neoplasms have measurable lesions according to Response Evaluation Criteria in Solid Tumors (RECIST v1.1) standard; (vi) not purged with other lavage fluid; (vii) voluntary participation.

The inclusion/exclusion criteria were as follows: (i) three months have passed after the end of radiotherapy; (ii) severe cardiac insufficiency and coronary heart disease; (iii) having a tendency to bleed and receiving anticoagulant or hemostatic drugs; (iv) severe erosion of nasopharynx and surrounding tissues, and intolerance to nasopharyngeal lavage fluid.

### Treatment

#### Nasopharyngeal lavage

**Figure 1:**
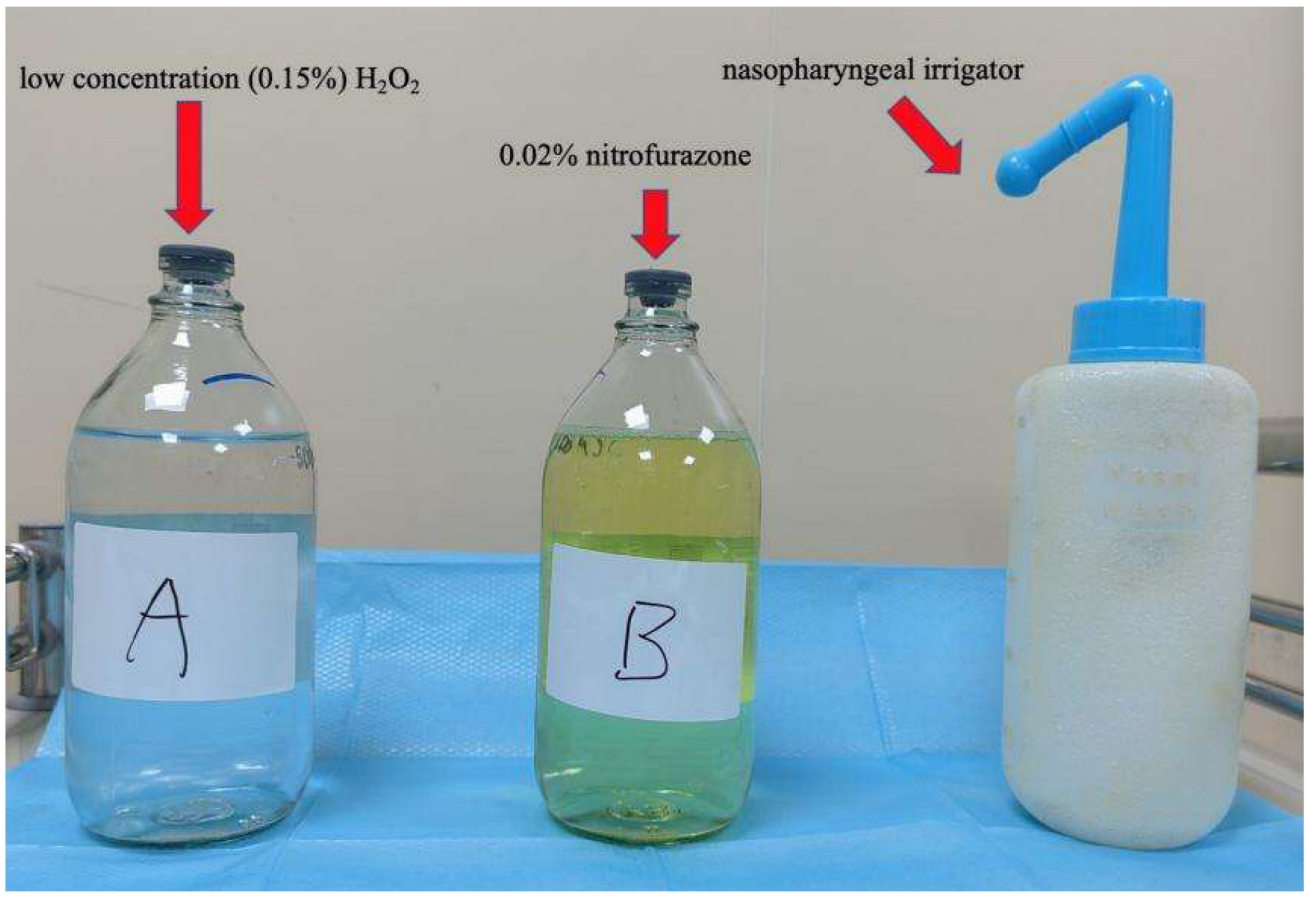
Nasopharyngeal lavage fluid of two groups and nasopharyngeal irrigator.

**Figure 2:**
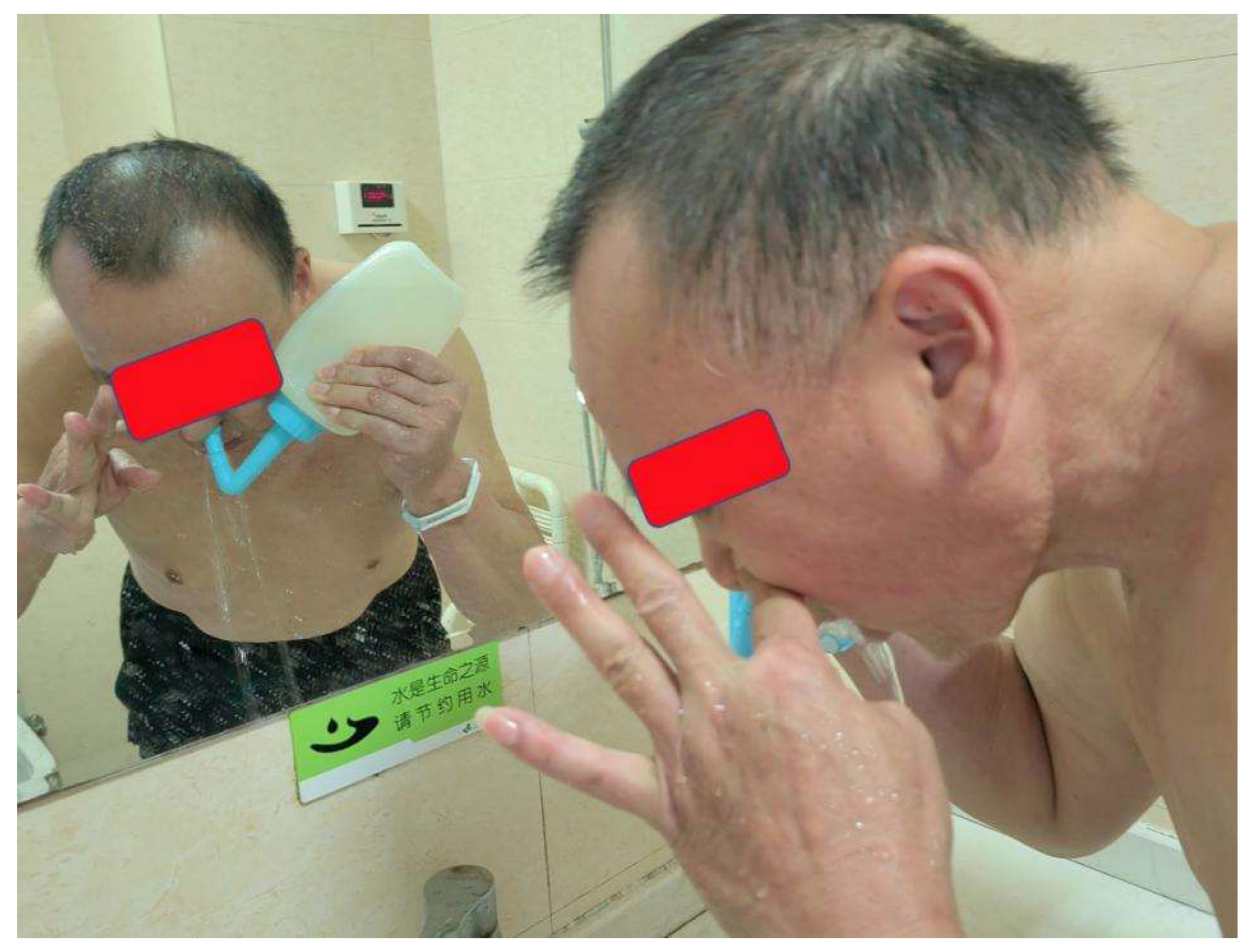
The purging method and position for NPC patient.

Configuration of the nasopharyngeal lavage fluid, training cohort: 3% medical H_2_O_2_ 25 ml was added into 475 ml Aqua Dest. After mixing the two liquids, the 0.15% concentration H_2_O_2_ was obtained, then heated to 37.0 ^0^C, and finally used twice a day after each day of radiotherapy; Control cohort: 0.02% nitrofurazone 500 mL, heated to 37.0 ^0^C, also to be used twice a day after each day of radiotherapy (**Figure 1**). The purging method consisted in having the patient take a sitting or standing position, lean their head slightly forward, and having the lavage fluid infused through the nasal cavity using the special nasopharyngeal irrigator, then spitted out through the mouth. During the lavage, the patient was instructed to breathe through the mouth. The left and right nasal passages were alternately washed using this method (**Figure 2**).

#### Radiotherapy

Patients received nasopharyngeal and/or neck lymph node region radiotherapy. They were placed in a supine position and their head, neck, and shoulder joints were fixed with a radiation mask. Magnetic resonance imaging (MRI) scanning technology was adopted, with a 3mm thickness for each layer. The scanning site range was from the top of head to the sternoclavicular joint. The images were subsequently transmitted to the Pinnacle TPS planning system. Target areas, neck lymph node region, and organs at risks (OARs) were identified according to reporting standards 50 and 62 of the International Commission on Radiation Units and Measurements (ICRU). The tumor and lymph node region were identified gross tumor volume (GTV_g_) and GTV_nd_. They were given 70 Gy and 66 Gy/ 33 fractions, respectively. The clinical target volume (CTV) was given 60 Gy/ 33 fractions, 5 times per week. Radiotherapy plan was used by Intensity Modulated Radiation Therapy (IMRT), while the energy of the linear accelerator was 6MV X ray (https://education.nccn.org).

#### Efficacy evaluation criteria

Short-term therapeutic efficacy imaging was centralized and reviewed by two radiologists. The responses were determined according to RECIST v1.1 10, including CR, PR, SD, and PD. The ORR was calculated as a ratio of the CR and PR for the patient population, ORR ratio was equal to the number of CR added to PR cases, then multiplied by 100%. While the disease control rate (DCR) was calculated as a ratio of the CR, PR and SD for the patient population. Efficacy evaluation imaging was performed by MRI scan plus enhancement scan.

#### Evaluation radiation toxicity criteria

According to the toxicity criteria of the Radiation Therapy Oncology Group (RTOG) (https://www.rtog.org) and the European organization for Research and Treatment of Cancer (EORTC) (https://www.eortc.org), acute radiation toxicity is classified by the following criteria. Grade 0: no change; Grade 1: congestion/mild pain, no analgesic required; Grade 2: flake inflammation, or inflammatory secretions, and moderate pain, analgesic required; Grade 3: fused fibrous flake inflammation, severe pain, requiring anesthesia; Grade 4: ulceration, hemorrhage and necrosis. Membrane reaction of nasopharynx and surrounding tissues was observed under electronic nasopharyngeal endoscopy.

#### Linear visual analogue scale

A questionnaire was used to classify patients according to their conscious feelings with the linear VAS score evaluation criteria. The specific classification was as follows: 0 score: no discomfort; 1 to 4 scores: mild discomfort; 5 to 7 scores: moderate discomfort; 8 to 10 scores: severe discomfort. All questionnaires were conducted after radiotherapy and nasopharyngeal lavage until therapeutic completion. The final value was calculated as an average of the results of three questionnaires administered at separate times.

### Statistical methods

The data were processed with SPSS statistics (Version 20.0; IBM Corp, USA). The results were presented as mean values and with 95% confidence intervals (95% CI). Fisher's exact test was used for difference analysis between two cohorts. Two-sided tests of statistical hypotheses with a P value < 0.05 were considered statistically significant.

## Results

### Patient characteristics

A total of 100 patients which had complete case data were recruited and analyzed from the Department of Head and Neck Radiotherapy of Jiangxi Cancer Hospital. All patients were randomly divided into two cohorts, one, the training cohort (n = 50), received the low concentration (0.15%) H_2_O_2_ as nasopharyngeal lavage fluid in the treatment for nasopharyngeal carcinoma radiotherapy. The control cohort (n = 50) received 0.02% nitrofurazone lavage fluid. The patient baseline characteristics were listed in **Table 1**.

### Efficacy

Comparison of short-term efficacy between the two cohorts. All patients were evaluated, including 38 (38.0%) CR, 48 (48.0%) PR, 14 (14.0%) SD and 86 (86.0%) ORR. There was no significant statistical difference between the training cohort and control cohort (*χ*^2^ = 1.432, P = 0.721) (**Table 2**). Comparison of linear VAS score evaluation between the two cohorts after purging of lavage fluid with nasopharyngeal patients. The training cohort scored significantly lower than the control cohort in linear VAS evaluation. There was a significant statistical difference between the two cohorts (*χ*^2^ = 13.988, P = 0. 003) (**Table 3**).

### Safety and Adverse events

About safety and AEs, all of the patients in the two cohorts had completed nasopharynx radiotherapy according to the original treatment schedule except for two patients in the control cohort, who could not tolerate the thrombocytopenia AEs of the concurrent chemoradiotherapy. All patients had different levels of radiation-related toxicity, including dermatitis, mucosa damage, bone pain, leukopenia, hemoglobin deficiency and thrombocytopenia. The incidence rate of Grade 3 toxicity or higher in the training and control cohort were 22.0%. 26.0%, 8.0%, 20.0%, 14. 0%, 18. 0% and 30.0%, 60.0%, 8.0%, 22.0%, 18.0% respectively. In all kinds of toxicities, there were no significant statistical differences between the two cohorts (P > 0.5). The nasopharyngeal mucosa damage in the training cohort was significantly lighter than that in the control cohort. The incidence rate of Grade 1 and 2 were 37 cases (74.0%) and 20 cases (40.0%). Compared with Grade 3 and 4 toxicity, the incidence in the control cohort was nonetheless higher than in the training cohort, namely 26.0% and 60.0%, respectively, which proved the existence of a significant statistical difference with two cohorts (*χ*^2^ = 14.679, P = 0.002) (**Table 4**).

## Discussion

According to the 2020 Global Cancer Statistics, in that year there were 133,354 newly diagnosed NPC cases, and almost 80,008 deaths from new cases 11. NPC has reportedly the highest morbidity of all tumors in Southern China, and radiotherapy plays a very important role in its treatment 12. Currently, concurrent chemoradiotherapy (CCRT) is the standard of treatment for locally advanced nasopharyngeal carcinoma (LANPC); CCRT can improve 3-years local control of nasopharyngeal carcinoma by 17.8% 13, 14. However, after radiotherapy, the nasopharynx is often affected by hyperemia, edema of membrane, and dermatitis of its surrounding tissues, especially under CCRT mode. Severe AEs can cause epithelial cells to die and fall off, which is usually followed by a proliferation of the local bacteria, leading to inflammation. With the present treatment modality of NPC, it is hard to improve the local control rate by optimizing the radiotherapeutic technology. Therefore, at present, the hot spots of research focus on how to carry out concurrent radiotherapy with other systemic therapies for NPC. Systemic therapy with immune checkpoint inhibitors and antiangiogenic agents has been suggested to improve local control and overall survival. However, the AEs of patients treated with CCRT are reported to increase significantly. Hyperemia and necrosis of nasopharyngeal mucosa is one of the AEs 15-18. The failure to timely discharge necrotic tissue of the nasopharyngeal tract will influence the radiation dose distribution and the sensitivity of cancer cells to radiotherapy, and in severe conditions, it may affect the therapeutic efficacy for NPC patients 5, 19. The nasopharyngeal lavage is one of the most important auxiliary treatments for NPC radiotherapy. Correct applications of nasopharyngeal lavage can improve the efficacy of NPC radiotherapy and reduce the incidence of AEs caused by radiotherapy 20. At present, there are a variety of nasopharyngeal lavage fluids used for patients in clinical practice, such as normal saline, potassium permanganate, medicinal H_2_O_2_, nitrofurazone fluid, sodium bicarbonate and Chinese traditional medicine 6, 21-23. However, the clinical efficacy of nasopharyngeal lavage is highly heterogeneous. Different lavage fluids and lavage methods are closely related to clinical efficacy. Past reports showed that H_2_O_2_ has the effect of clearing necrotic tissue and preventing the spread of anaerobic bacteria 1, 6, 19, 24. However, the efficacy of H_2_O_2_ as nasopharyngeal lavage fluid for the treatment with NPC patients has not been accurately reported, and it is still unclear whether it is relevant to improve the efficacy of NPC and reduce the AEs of radiotherapy.

In this study, a paired cohort was used to evaluate and analyze the efficacy and safety of low concentration (0.15%) H_2_O_2_ as a nasopharyngeal lavage fluid in the auxiliary treatment of NPC patients. There were 38 (38.0%) CR, 48 (48.0%) PR, 14 (14.0%) SD and 86 (86.0%) ORR for whole cohort NPC patients with radiotherapy or CCRT. However, the results indicated that the training cohort did not experience any significant improvement in terms of efficacy compared to the control cohort, which instead received nitrofurazone fluid with short-term efficacy. Statistical analysis showed no significant difference between the training cohort and the control cohort (P = 0.721).

**Figure 3:**
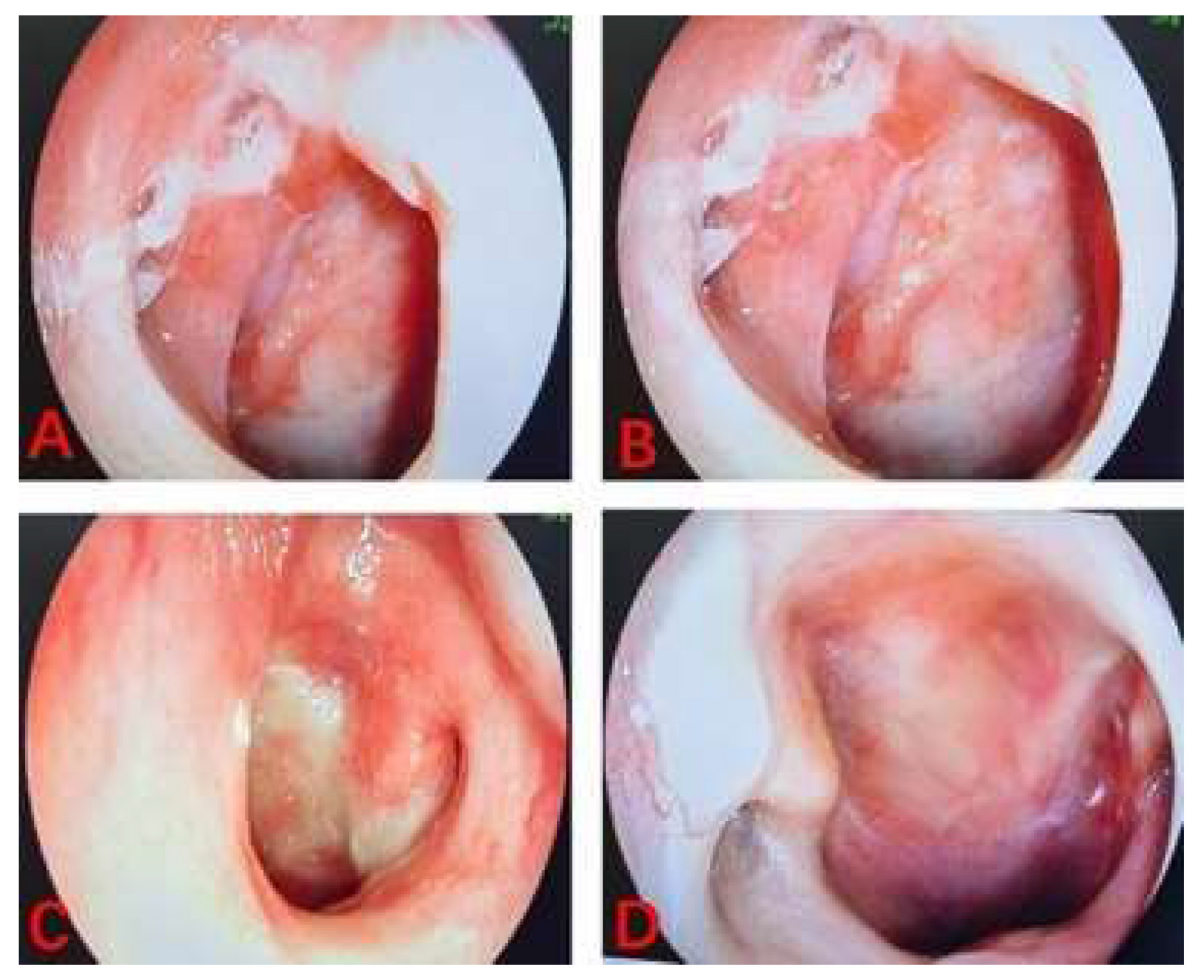
The images of process of nasopharyngeal lavage for NPC patient in training cohort.

**Figure 4:**
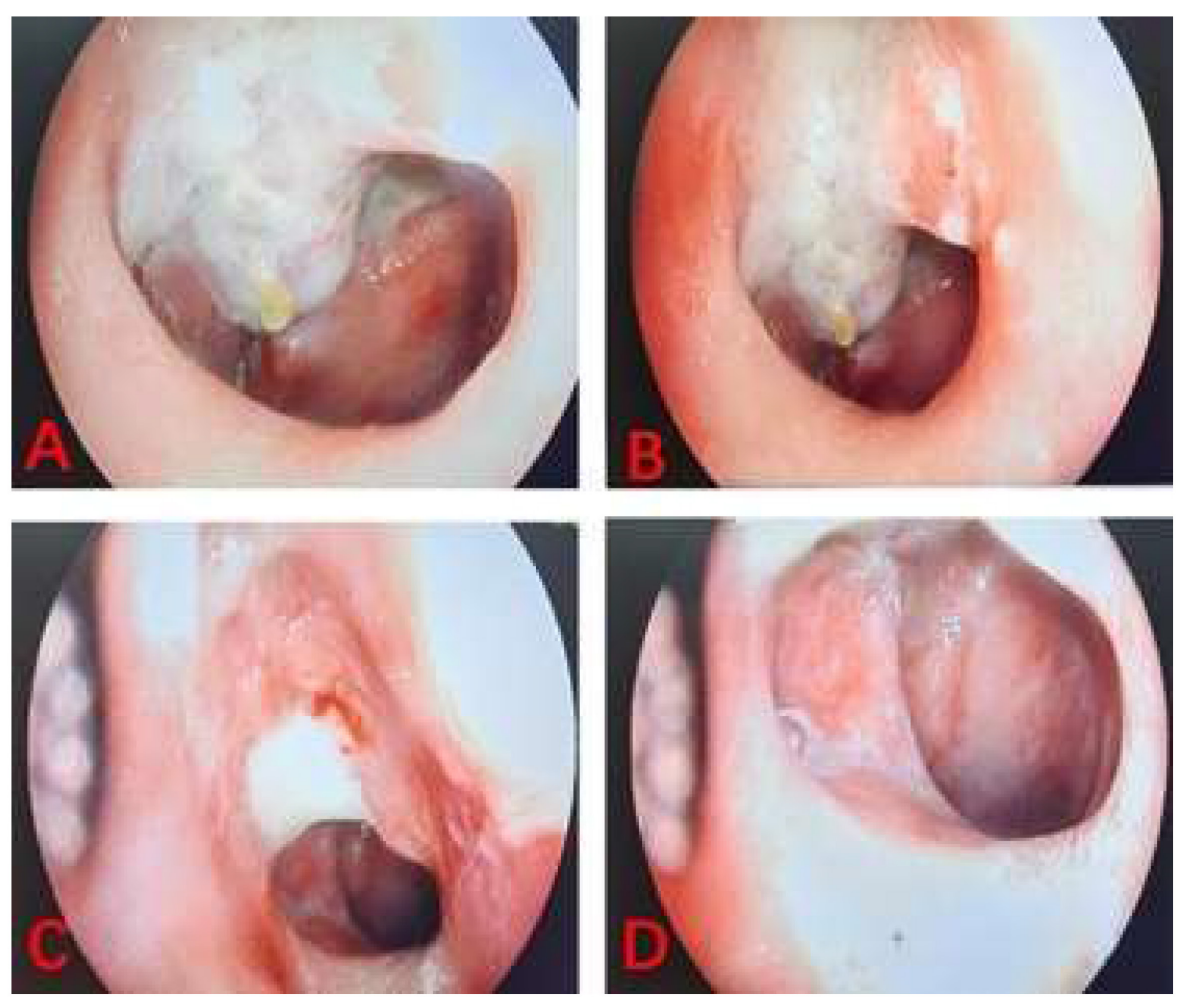
The images of process of nasopharyngeal lavage for NPC patient in control cohort.

The linear VAS score of the training cohort was lower than that of the control cohort with nasopharyngeal lavage after nasopharyngeal radiotherapy. According to the results of the linear VAS score, feelings of comfort in NPC patients belonging to the training cohort was significantly better than those recorded in patients in the control cohort. The two images shown the process of nasopharyngeal lavage for two different patients in training cohort (**Figure 3**) and control cohort (**Figure 4**), respectively. The difference between the two cohorts was shown to be statistically significant (P = 0.003). It is worth to mention that all patients completed their prescribed treatment plan in the training cohort, while in the control cohort, two patients gave up the treatment because of serious radiation-related thrombocytopenia AE. In summary, the results suggested that low concentration (0.15%) H_2_O_2_ as a nasopharyngeal lavage fluid is a viable auxiliary treatment, and its main contribution to radiotherapy with NPC patients is to reduce the AEs of radiotherapy, perfect the comfort of patients, and improve patient adherence to treatment, despite not having any decisive effect on the curative efficacy of radiotherapy.

At present, the main factors that are considered to be related to the curative efficacy are still radiotherapy and chemotherapy techniques, clinical stages and pathological types. In terms of safety and AEs, this study showed that all patients in the two cohorts experienced different grades of radiation-related toxicity, mainly including dermatitis, mucosa damage, bone pain, leukopenia, hemoglobin deficiency and thrombocytopenia. There was no significant statistical difference between the two in all Grades or Grade 3 and 4 except mucosa damage. Further analysis showed that nasopharyngeal mucosa damage in the training cohort was significantly less than that in the control cohort, with 37 cases (74.0%) and 20 cases (40.0%) of Grade 1 or 2, respectively. In terms of Grade 3 and 4 toxicity, the control cohort recorded a higher number of instances than the training cohort. Namely, these were reported to be 26.0% and 60.0%, respectively. These results better explained why patients in the training cohort experienced better comfort compared with those in the control cohort. The reason was that low concentration (0.15%) H_2_O_2_ as a nasopharyngeal lavage fluid can effectively remove necrotic tissue and inflammatory secretions, and improved patients' conscious comfort after nasopharyngeal radiotherapy.

## Conclusion

The application of low concentration (0.15%) H_2_O_2_ as nasopharyngeal lavage fluid for NPC patients with radiotherapy is feasible and safe, it can improve comfort in NPC patients, and reduce local radiation related mucosa toxicity after receiving radiotherapy. Due to the limited sample size of this study, the research results still need to be confirmed by studies involving large samples and multi-center research. Long-term follow-up is needed to assess disease free survival and overall survival with nasopharyngeal lavage fluid auxiliary treatment.

## Figures and Tables

**Table 1:** Patient clinical characteristics of the training and control cohorts.

Characteristic	Training cohort (n)	Control cohort (n)	*χ* ^2^	*P*
	n= 50	n= 50		
Years			0.372	0.542
≥ 60	19	22		
< 60	31	28		
Gender			0.198	0.656
Male	15	13		
Female	35	37		
Clinic stage			1.143	0.887
I	3	4		
II	12	13		
III	26	21		
IV_A_	6	8		
IV_B_	3	4		
T stage			3.419	0.331
T_1_	6	8		
T_2_	13	18		
T_3_	24	15		
T_4_	7	9		
N stage			0.803	0.849
N_0_	9	11		
N_1_	23	19		
N_2_	10	10		
N_3_	8	10		
Concurrent chemoradiotherapy			0.184	0.668
Yes	35	33		
No	15	17		
ECOG score			1.169	0.280
2	13	18		
<2	37	32		
Using antibiotics			0.407	0.523
Yes	35	32		
No	15	18		

**Table 2:** Comparison of therapeutic effect between two cohorts after radiotherapy (n).

Cohorts	CR	PR	SD	ORR	*χ* ^2^	*p*
Training	18	23	9	41		
Control	20	25	5	45		
Total	38	48	14	86	1.432	0.721

**Table 3:** Comparison of VAS scores between the two cohorts.

Cohorts	0 score	1~ 4 scores	5~ 7 scores	8~ 10 scores	*χ* ^2^	*p*
Training	17	20	8	5		
Control	5	15	18	12	13.988	0.003

**Table 4:** Comparison of radiation-related toxicity after radiotherapy between two cohorts.

Adverse events	Training cohort				Control cohort				*χ* ^2^	*p*
	1	2	3	4	1	2	3	4		
Dermatitis	11	28	10	1	6	29	13	2	2.213	0.529
Mucosa damage	13	24	12	1	3	17	25	5	14.679	0.002
Bone pain	42	6	2	0	38	8	4	0	1.152	0.562
Leukopenia	25	15	6	4	20	19	6	5	1.137	0.768
Hemoglobin deficiency	28	15	7	0	23	18	7	2	2.763	0.430
Thrombocytopenia	30	11	6	3	25	16	5	4	1.614	0.656
